# Biocompatibility and antibacterial properties of medical stainless steel and titanium modified by alumina and hafnia films prepared by atomic layer deposition

**DOI:** 10.1007/s10856-024-06841-8

**Published:** 2024-11-12

**Authors:** Ivan Spajić, Miguel Gonçalves Morais, Cláudia Monteiro, M. Cristina L. Martins, Ana Paula Pêgo, Ingrid Milošev

**Affiliations:** 1 Jožef Stefan Institute, Department of Physical and Organic Chemistry, Jamova c. 39, Ljubljana, SI-1000 Slovenia; 2 Jožef Stefan International Postgraduate School, Jamova c. 39, Ljubljana, SI-1000 Slovenia; 3 i3S – Instituto de Investigação e Inovação em Saúde, Universidade do Porto, Rua Alfredo Allen, 208, 4200-135 Porto, Portugal; 4 INEB – Instituto de Engenharia Biomédica, Universidade do Porto, Rua Alfredo Allen, 208, 4200-135 Porto, Portugal; 5 ICBAS – Instituto de Ciências Biomédicas Abel Salazar, Universidade do Porto, R. Jorge de Viterbo Ferreira 228, 4050-343 Porto, Portugal; 6 Valdoltra Orthopaedic Hospital, Jadranska c. 31, Ankaran, SI-6280 Slovenia

## Abstract

New methods for producing surfaces with suitable biocompatible properties are desirable due to increasing demands for biomedical devices. Stainless steel 316 L and cp- titanium specimens were coated with thin films of alumina and hafnia deposited using the atomic layer deposition method at two temperatures, 180 and 260 °C. The morphology of the films was analysed using scanning electron microscopy, and their surface energies were determined based on drop contact angle measurements. Biocompatibility assays performed using mesenchymal stem cells were evaluated by incubating the specimens and then exposing their extracts to the cells or directly seeding cells on the specimen surfaces. No detrimental effect was noticed for any of the specimens. Antibacterial properties were tested by directly incubating the specimens with the bacteria *Staphylococcus aureus*. Overall, our data show that all prepared films were biocompatible. Alumina films deposited on cp-titanium at 260 °C outperform the other prepared and tested surfaces regarding antiadhesive properties, which could be related to their low surface energy.

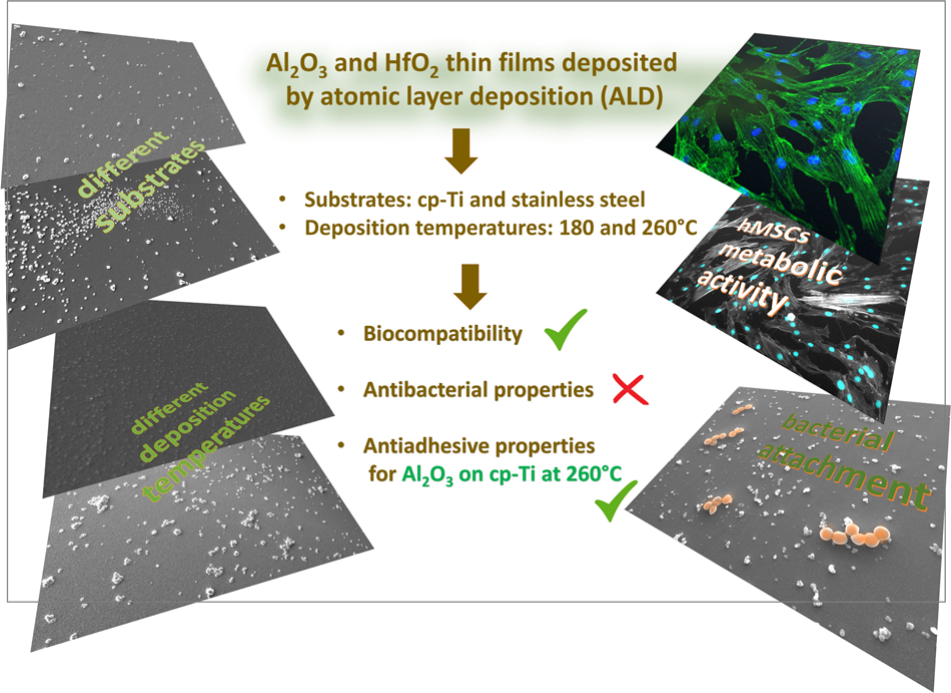

## Introduction

The main characteristics of metallic biomaterials are suitable mechanical properties, chemical inertness and biocompatibility with the surrounding in vivo environment [1, 2]. Although alloys used in biomedicine, such as stainless steel and titanium alloys, are aligned with all these requirements, one of the critical causes of failure of implantable medical devices is an infection, which is directly related to the formation of bacterial biofilms on the implant’s surface [3, 4]. The first critical factor in bacteria attachment and biofilm formation is also dependent on processes of protein adsorption that lead to the rapid formation of a thin layer of proteins at the implant’s surface after implantation [5]. Therefore, the implant surface should possess antibacterial properties, which implies the ability of a surface to resist the initial attachment of bacteria by either exhibiting an antiadhesive or bactericidal effect. Antiadhesive surfaces may resist bacterial attachment due to specific surface physical and chemical properties unfavourable for bacteria attachment. Further, bactericidal surfaces may disrupt the physiological functions of bacteria, causing their death by releasing chemical agents interrupting the bacterial growth cycle or inducing bacterial killing [6–8].

On the other hand, cellular adhesion is often desirable, especially in implants used as scaffolds for tissue growth. Thus, the ideal implant surface should inhibit bacterial adhesion while promoting or even selectively promoting cellular adhesion, depending on the application [9]. Essentially, the response of implant material in the biological environment depends on surface roughness or topography, chemical composition and surface energy. The latter is directly related to the surface’s wettability and may affect the in vitro and in vivo behaviour of the implant material [10].

Medical stainless steel 316 L (SS316L) and commercially pure titanium (cp-Ti), which are used as substrates in this work, have good corrosion resistance in the biological environment [1, 11] and are usually considered to be materials of good biocompatibility [12, 13]. Although titanium alloys and SS316L are highly corrosion resistant due to the formation of highly protective passive films, in some cases, these alloys can be subject to localised corrosion attack in vivo [2]. This corrosion process may lead to the release of metal ions, such as allergenic aluminium (Al) and vanadium (V) [14–16] or potentially toxic nickel (Ni) and chromium (Cr) [15]. Therefore, different techniques aim to improve corrosion resistance and biocompatibility properties and prevent metal release in the periprosthetic environment [4, 17]. One of the ways to modify the surfaces of metals for implantation is by depositing a thin coating or film over the surface. In this way, the release of harmful metal ions into the surrounding tissue can be effectively prevented, and a favourable interaction of the implant with the surrounding tissue can be encouraged. For this purpose, different thin film deposition techniques are used: ion beam deposition [18], chemical vapour deposition (CVD) [19], pulsed laser deposition (PLD) [20], electrodeposition [21], etc. [2, 17]. The most deposited material is synthetic calcium hydroxyapatite, which simulates the naturally formed calcium phosphate in the human body and has excellent biocompatibility [22]. However, other ceramic materials that can provide barrier protection on the implanted material are of great interest as well [23]. Some of those ceramic materials are alumina (Al_2_O_3_) [24], titania (TiO_2_) [25], zirconia (ZrO_2_) [26], titanium nitride (TiN) [27], zirconium nitride (ZrN) [28], silicon nitride (Si_3_N_4_) [29], titanium-niobium nitride (TiNbN) [30] and diamond-like carbon (DLC) [31, 32].

One technique still in the early research phase for implant applications which offers the deposition of various ceramic materials is atomic layer deposition (ALD) [33]. The peculiarities of this technique are the ability to form thin, nanometric films with high thickness uniformity and conformity on very complex nano-shaped surfaces or even inside nano-porous materials [34, 35]. This feature can be important for some applications in biomedical engineering and biotechnology [36]. Another feature of ALD is the possibility of forming defect- and impurity-free films, which is crucial for barrier properties and corrosion protection [37]. Due to the above features, it is increasingly considered a technique for various applications in medicine and biotechnology, e.g. biosensors and diagnostics, biotemplating and protection of small implants for cardiovascular or cochlear systems [36, 38]. ALD-coated materials aim to modify the substrate’s surface to impart biologically relevant surface properties such as biocompatibility, bioactivity and antibacterial properties [38, 39].

In our previous studies, we addressed the morphology, composition and electrochemical properties of ALD alumina (Al_2_O_3_) and hafnia (HfO_2_) hafnia thin (less than 150 nm) films deposited on cp-Ti and SS316 at 160 °C and 180 °C, respectively [40, 41]. Both films are homogeneous and provide barrier protection of underlying substrates, with hafnia showing superior properties to alumina during long-term immersion in simulated physiological solution [40]. Therefore, the first step toward potential applications in biomedicine is to aim primarily for specific non-loaded parts of implants, such as cardiovascular or cochlear implants, due to the susceptibility of nanometric thin films to mechanical damage. The present study deals with the next step, i.e. testing the biocompatibility and antibacterial properties of ALD alumina and hafnia thin films.

Literature data on these issues, especially for hafnia, are scarce, which was an additional incentive for this study. For example, alumina is used in bulk form for femoral head hip replacement due to its favourable biomechanical and biocompatible properties [42, 43]. In addition, due to the favourable cellular response when interacting with biological tissue, the application of alumina as an implant material is intensively researched and plays a key role in biomedical engineering [43]. Alumina obtained as a film by ALD at temperatures below 600 °C is amorphous and can have significantly different properties than in the bulk form [44]. Therefore, there was a need to investigate alumina thin films obtained by the ALD technique for biomedical applications. Several studies have worked on this topic and confirmed the favourable biocompatible properties of ALD alumina thin films. Thus, Finch et al. investigated ALD alumina thin film in interaction with human coronary artery smooth muscle cells and reported good cell adhesion and growth [45]. Liang et al. showed accelerated development of hydroxyapatite and good adhesion of fibroblast cells on ALD alumina and titania-modified polymer particles [46]. Regarding the antibacterial properties of ALD alumina, there is only one short paper reporting the antibacterial activity of several ALD oxide films, including alumina [47]. Besides, studies on the modification or co-deposition of ALD alumina film with bactericidal materials, such as ZnO [48, 49], and many other ALD oxide films have been studied to obtain an antibacterial surface [50]. In general, it is known that alumina as a bulk material does not have a harmful effect on bacteria, while in the form of nanoparticles, it is known as an antimicrobial agent [51]. This is a consequence of the large specific surface, which ensures a broad range of reactions with bio-organics on the cell surface [52].

Hafnia has not been used for implantation and has rarely been explored for biomedical applications [53]. Additionally, there are several studies on Hf as an alternative metal in biomedical applications; it is much heavier but chemically very similar to Ti and analogously passivated with a protective layer of the hafnia [54, 55].

In the present study, ALD films were deposited at 180 °C and 260 °C, which gives different roughness due to different film growth kinetics [56–58]. Surface roughness at the micro and nano level is known to benefit the osseointegration of the implanted material [59–61]. Further, hafnia changes in crystallinity depending on the temperature [57], which can also be expected to affect the interaction with living cells and bacteria [61]. The morphology of the films was analysed using scanning electron microscopy (SEM). Their biocompatibility was assessed by exploring human bone marrow-derived mesenchymal stem cells as in vitro model. Antibacterial properties were tested against *Staphylococcus aureus*.

## Experimental part

### Specimens preparation

SS316L and cp-Ti specimens in the shape of a disc of 2.0 mm in thickness and 15 mm in diameter, supplied by GoodFellow Cambridge Ltd., were used as substrate materials. The cp-Ti was grade 2, with a purity of 99.6%, and SS316L with the composition of the main alloying elements as follows: 18 wt.% Cr, 10 wt.% Ni and 3 wt.% Mo, rest Fe, as reported by the supplier.

Both materials were polished until a mirror-like surface appearance (LaboPol-5, Struers). The polishing procedure consisted of two steps: grinding with 500-grit SiC emery papers (Struers, Denmark), followed by polishing with silica (SiO_2_) suspension (OP-S, Struers) with a particle size of 0.25 μm, with the addition of the chemical reagents, 30% H_2_O_2_ and 25% NH_4_OH (Merck KGaA). Following the polishing step, specimens were cleaned in 99.6% ethanol (Merck KGaA) using an ultrasonication bath Elmasonic P series (Elma Schmidbauer GmbH) for 15 min and then dried with high-pressure nitrogen gas. All specimens were stored overnight (ca. 16 h) in a vacuum desiccator filled with commercial hygroscopic silica gel to ensure uniform surface conditions before the ALD deposition processes.

Pure copper (Goodfellow, Cambridge, UK), known as a bactericide, was used as a positive reference specimen to test antibacterial properties. It was prepared by grinding with SiC emery papers up to 4000 grit, then polishing with the OP-S suspension of 0.25 μm particle size.

### ALD deposition processes

A TFS 200 system by Beneq Oy was used to deposit alumina (Al_2_O_3_) and hafnia (HfO_2_) thin films in the cross-flow reactor [33]. Alumina was deposited using trimethylaluminium (Al(CH_3_)_3_ or TMA, 99.99% PURATREM, STREM Chemicals Inc.) as the first precursor. Hafnia was deposited using tetrakis(ethylmethylamido)hafnium(IV) (Hf[N(CH_3_)(C_2_H_5_)]_4_ or TEMAH, 99.99% PURATREM, STREM Chemicals Inc.) as the first precursor. Milli-Q water (resistivity 18 MΩ·cm at 25 °C, Billerica, MA) was used as the second precursor for both types of thin films. For alumina, the ALD cycle consisted of a 0.35 s TMA dose, a 1 s N_2_ purge, a 0.3 s water dose and a 1 s N_2_ purge, and for the hafnia, it consisted of a 0.5 s TEMAH dose, a 1 s N_2_ purge, a 0.2 s water dose and a 1 s N_2_ purge. The expected growth rate per cycle (GPC) for alumina deposition was 1 Å/cycle, as reported in previous work [62] for similar ALD conditions. According to previous work, the expected GPC for hafnia is around 0.9 Å/cycle [63]. The target thickness of both alumina and hafnia films was 60 nm, so 600 cycles of ALD deposition were performed for alumina and 670 cycles for hafnia. Therefore, the expected thickness of the films is 60 nm, noting that it is known from our previous work that thicknesses can vary by several nanometers [40]. However, the thicknesses of the films were not specifically measured for this research because the focus was exclusively on the surface of the films. Both described ALD procedures were performed at 180 °C and 260 °C. The designation of specimens is presented in Table 1.

**Table 1:** Designation of investigated specimens used in this study

Specimen	Designation	Specimen	Designation
commercially pure Ti	cp-Ti	stainless steel 316 L	SS316L
cp-Ti coated with ALD alumina deposited at 180 °C	Ti-AL180	SS coated with ALD alumina deposited at 180 °C	SS-AL180
cp-Ti coated with ALD alumina deposited at 260 °C	Ti-AL260	SS coated with ALD alumina deposited at 260 °C	SS-AL260
cp-Ti coated with ALD hafnia deposited at 180 °C	Ti-HF180	SS coated with ALD hafnia deposited at 180 °C	SS-HF180
cp-Ti coated with ALD hafnia deposited at 260 °C	Ti-HF260	SS coated with ALD hafnia deposited at 260 °C	SS-HF260

### Surface characterisation

#### Surface morphology

Morphological characterisation of the specimen surfaces was performed using a scanning electron microscope (SEM), a JEOL JSM-7600 F. Analyses were performed at beam acceleration voltages at 2 kV and 3 kV using an Everhard-Thornley (ETD) detector for secondary electrons to obtain insights into the morphology of the surfaces. Magnifications between 5,000× and 20,000× were used to obtain the surface features on the micrometre scale.

#### Wettability and surface energy

The quantitative measure of the wetting of a solid by a liquid is the contact angle, *θ*, which is the angle formed by a liquid at the three-phase boundaries where a liquid, a gas and a solid intersect. In this work, the surface energies of the prepared specimens were determined by measuring the contact angles of liquid drops on the surfaces. The Fowkes model, which employs two different liquids, one polar and another non-polar, to measure the contact angle (CA), was used [64, 65]. Water was used as a polar liquid and diiodomethane as a non-polar, with similar experimental conditions as reported [65]. In the Fowkes model, the dispersive component of the solid surface energy, ${\gamma }_{{\rm{s}}}^{{\rm{d}}}$, the polar component of the solid surface energy, ${\gamma }_{{\rm{s}}}^{{\rm{p}}}$, and the total solid surface energy, ${\gamma }_{{\rm{s}}}$, are determined by Eqs. (1) and (2):

1 $${\gamma }_{{\rm{s}}}={\gamma }_{{\rm{s}}}^{{\rm{d}}}+{\gamma }_{{\rm{s}}}^{{\rm{p}}}$$

2 $${\gamma }_{{\rm{l}}}\bullet \left(1+\cos \varTheta \right)=2\left(\sqrt{{\gamma }_{{\rm{l}}}^{{\rm{d}}}\bullet {\gamma }_{{\rm{s}}}^{{\rm{d}}}}+\sqrt{{\gamma }_{{\rm{l}}}^{{\rm{p}}}\bullet {\gamma }_{{\rm{s}}}^{{\rm{p}}}}\right)$$

where *θ* is the measured contact angle between the solid and the liquid; ${\gamma }_{{\rm{l}}}$ is the total surface energy of the test liquids (${\gamma }_{{\rm{l}}}$ = 72.8 mJ/m^2^ for water and ${\gamma }_{{\rm{l}}}$ = 50.8 mJ/m^2^ for diiodomethane); ${\gamma }_{{\rm{l}}}^{{\rm{d}}}$ is the liquid’s dispersive component (${\gamma }_{{\rm{l}}}^{{\rm{d}}}$ = 21.8 mJ/m^2^ for water and ${\gamma }_{{\rm{l}}}^{{\rm{d}}}$ = 50.8 mJ/m^2^ for diiodomethane); and ${\gamma }_{{\rm{l}}}^{{\rm{p}}}$ is the liquid’s polar component (${\gamma }_{{\rm{l}}}^{{\rm{p}}}$ = 51.0 mJ/m^2^ for water and ${\gamma }_{{\rm{l}}}^{{\rm{p}}}$ = 0 mJ/m^2^ for diiodomethane). Thus, testing two liquids yields two equations with two unknowns, dispersive and polar solid components, giving total solid surface energy Eq. [1].

The measurement of the static contact angle was carried out through the sessile drop method. A series of at least five drops (4 μl/drop) were used on each type of specimen. The contact angles of drops of deionised water and diiodomethane were measured using the Krüss EasyDrop DSA 20E instrument equipped with DropShape software. The resolution of the instrument was 0.1°. The baseline was fitted manually, and the program calculated the contact angle.

The contact angle measurements were performed on all specimens immediately after surface preparation and after the deposition of thin films, respectively. After surface sterilisation, the measurements were also carried out to check whether this procedure affects specimens’ wettability and surface energy.

### Biocompatibility

Biocompatibility assays were performed in two ways: [1] by incubating the specimens and using their extracts and [2] by directly seeding the cells on the specimen surfaces. Specimens were sterilised by immersion in serial dilutions of ethanol (90, 70, and 50% (v/v)) and rinsed with autoclaved deionised water followed by phosphate-buffered solution (PBS) for 15 min. Human bone marrow-derived mesenchymal stem cells (hMSCs - Lonza) were used to perform both assays. Unless mentioned otherwise, all reagents were obtained from Sigma in analytical grade.

#### Assays with extracts

**Scheme 1:**
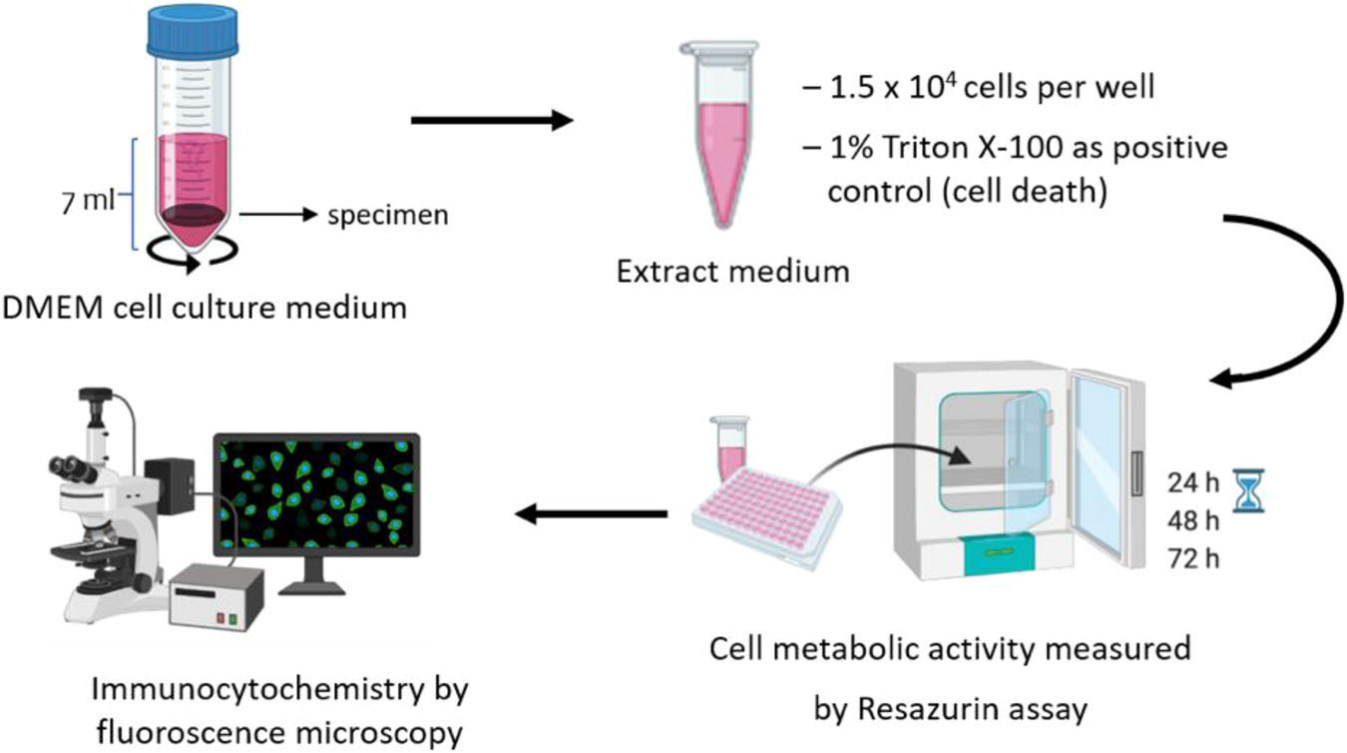
Schematic representation of the performed assays by using extracts. Cell metabolic activity was followed for the same cultures as a function of time. Cell morphology was assessed at the experimental endpoint

Extracts were obtained by placing each specimen in 7 mL of Dulbecco’s modified eagle medium (DMEM – 21885025 Thermo Fisher Scientific) with low glucose supplemented with 1% (v/v) penicillin-streptomycin (15070063 – Thermo Fisher Scientific) and incubating overnight in an orbital incubator at 37 °C and 180 rpm. The following day, specimens were removed, and the extracts were supplemented with 10% (v/v) fetal bovine serum (FBS – 10094563 Thermo Fisher Scientific) previously inactivated by heat (30 min at 56 °C). Cells were seeded at a density of 1.5 × 10^4^ viable cells/cm^2^ (as determined by the trypan blue assay) in 96-well tissue culture plates (353072 - Falcon) with 3 replicates for each sample type, subsequently treated with the extracts and kept at 37 °C in a humidified atmosphere of 5% CO_2_ for up to 72 h. Scheme 1 shows an overview of the experimental setup.

#### Test by direct contact

Specimens sterilised as previously described (see Section 2.4.1) were placed in 12-well non-treated plates (150200 - Nunc). Cells were seeded directly on specimens at a density of 1.1 × 10^4^ viable cells/cm^2^ (as determined by the trypan blue assay) and maintained in culture for 7 days, with the medium being changed every other day.

#### Resazurin assay

To monitor cell metabolic activity and infer cell viability, a resazurin assay was performed for cells in contact with material extracts or metal specimens. Resazurin is a blue, non-fluorescent molecule that several enzymes reduce to a pink, fluorescent product called resorufin. The amount of resorufin produced is directly proportional to the number of metabolically active cells. A resazurin solution (0.1 mg/mL in PBS) was added to each well to a final concentration of 10% (v/v). After 3 h of incubation at 37 °C, 200 µL of the medium was transferred into a 96-well black-walled plate, and fluorescence was measured at excitation and emission wavelengths of 530 and 590 nm, respectively (Synergy Mx microplate reader – Biotek Instruments). A 1% (v/v) solution of Triton X-100 (BP151-500 – Sigma Aldrich) prepared in a fresh culture medium was used as a positive control (cytotoxic agent).

#### Immunocytochemistry

To assess cell morphology, at the end of the experimental period (3 days for extracts and 7 days for direct contact), the cell culture medium was removed, and cells were fixed with 4% (w/v) p-formaldehyde (30525-89-4 – TCI) in PBS for 15 min. Cytoskeletal filamentous actin (F-actin) was stained by incubating cells with Alexa Fluor 488 phalloidin (a12379 - Invitrogen), 5 U/mL, for 30 min in the dark. Cells were washed thrice with PBS, and cell nuclei were counterstained with Hoescht 33342 (H3570 - Invitrogen). Samples were imaged with an inverted epifluorescence microscope (Leica DMI6000 FFW)

### Antibacterial properties

Antibacterial properties were assessed by incubating the specimens with the bacteria *Staphylococcus aureus* (ATCC 25923). First, all prepared specimens were sterilised twice by immersion in 70% (v/v) ethanol for 15 min, then rinsed three times with sterile Milli Q water, dried with argon and stored in a 24-well plate saturated with argon. A drop (35 µL) of bacterial inoculum at a concentration of 1 × 10^7^ colony-forming units (CFU/mL) was placed on the surface of the specimen and covered with a polypropylene film. Specimens were then placed in a 24-well plate inside a humidified box to avoid evaporation and left to incubate at 37 °C for 2 h. After incubation, the polypropylene film was removed, and 1 mL of sterile PBS was added to each sample, i.e. the contents in incubation media were collected and diluted for CFU counting. Then, specimens were rinsed three times with PBS and prepared for SEM analysis to visualise adherent bacteria or processed to detach adherent bacteria by sonication.

To detach adhered bacteria, a protocol described here [7] was followed. Specimens were transferred to 50 mL Falcon tubes containing 1 mL of 0.5% (v/v) Tween 80 in PBS and then sonicated (BactoSonic®, BANDELIN) at 160 W for 15 min, placed on ice for 5 min and vortexed, and sonicated again for 15 min. Then, serial dilutions were prepared and plated for CFU counting of surface adherent bacteria.

#### Scanning electron microscopy of samples exposed to bacteria

Specimens were prepared according to a procedure described here [66]. After collecting content in incubation media, specimens were washed three times with PBS, as described above. The adherent bacteria were then fixed with a freshly prepared solution of 1.5% glutaraldehyde in 0.14 M of sodium cacodylate buffer for 30 min at room temperature. After fixation, the specimens were rinsed twice with deionised water and dehydrated using a growing ethanol/water gradient (50%, 60%, 70%, 80%, 90% and 99% (v/v)), maintaining the specimens in each solution for 10 mins. Then, 1 mL of hexamethyldisilazane was added to each specimen, and they were left to dry overnight. The specimens were placed onto SEM pin stubs (TED PELLA, Inc., USA) using carbon tape and sputtered with an Au/Pd thin film (15 mA, 60 s) to improve their conductivity. The bacteria morphology and distribution were characterised by SEM. In addition, the surface topography of the as-deposited specimens was characterised to find a possible correlation with biocompatibility or antibacterial properties. SEM Quanta 400 FEG ESEM / EDAX Genesis X4M (ThermoFischer, USA) was used.

### Statistical analysis

#### Resazurin assay

Before statistical analysis, data was normalised (log transformation) and tested for normality. Data was analysed using 2-way repeated measures mixed model approach, with specimens as the treatment factor, day in vitro as the repeated factor and the experience number as the blocking factor. This was followed by planned comparisons on the predicted means to compare the levels of the effect Specimens * Day in vitro. A full description of the mixed model theory can be found in the following reference [67]. Statistical analysis was performed with InVivoStat, version 4.4.

#### Antimicrobial properties

Statistical analysis was performed using GraphPad Prism version 9.0.2 for Mac OS X (GraphPad Software, California, USA). Ordinary one-way analysis of variance (ANOVA) with Dunnett’s multiple comparisons test was used to compare multiple data groups to control.

#### Wettability

Statistical analysis of contact angle results was performed in Microsoft Excel by calculating standard deviation and t-test. The t-test was performed to determine the statistical significance of the difference in wettability between ALD-coated specimens and cp-Ti and SS316L specimens, respectively.

## Results and discussion

### Surface morphology

**Fig. 1:**
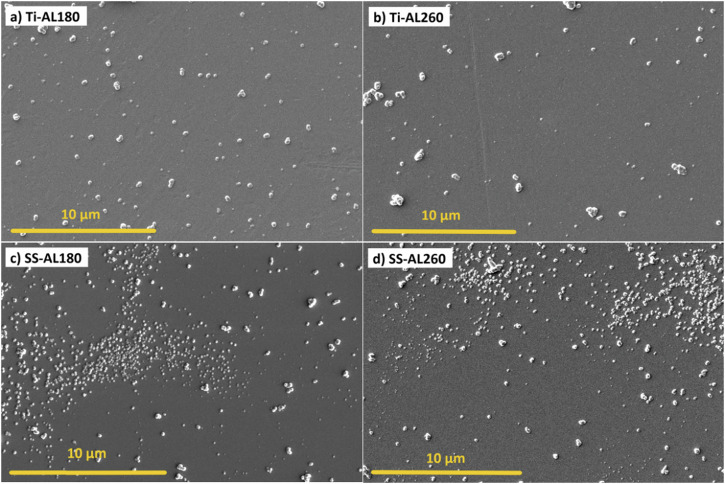
SEM images of ALD alumina-coated cp-Ti and SS316L specimens for **a** cp-Ti coated at 180 °C, **b** cp-Ti coated at 260 °C, **c** SS316L coated at 180 °C and **d** SS316L coated at 260 °C

The surface morphologies of cp-Ti and SS316L specimens coated with ALD alumina thin films deposited at different temperatures, 180 °C and 260 °C, are shown in Fig. 1. The presence of agglomerates, sized around 1 µm, randomly distributed over the surface can be observed in all specimens. Additionally, alumina agglomerates on SS316L substrate appear to form preferentially in some areas and with a tendency to form very small, densely distributed agglomerates below 1 µm in size (Fig. 1c, d). The distribution of agglomerates seems independent of deposition temperature; i.e. at both temperatures, the surface morphologies are approximately equal.

**Fig. 2:**
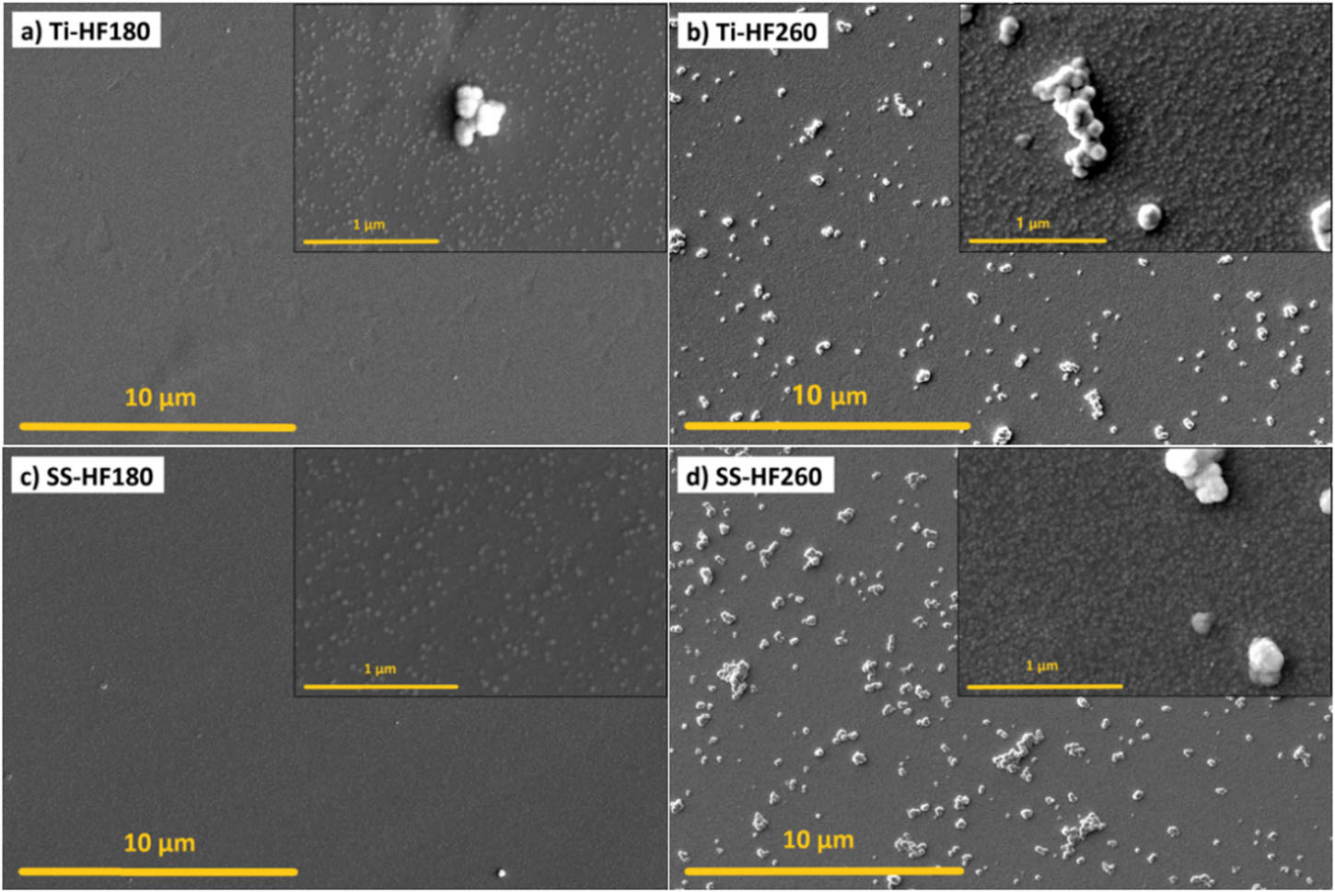
SEM images of ALD hafnia-coated cp-Ti and SS316L specimens for **a** cp-Ti coated at 180 °C, **b** cp-Ti coated at 260 °C, **c** SS316L coated at 180 °C, and **d** SS316L coated at 260 °C

ALD hafnia thin films exhibit different surface morphology than alumina films. Namely, the presence of agglomerates depends strongly on the deposition temperature (Fig. 2). When deposited at 180 °C, the surface of the hafnia thin films appears uniform on a given scale (Fig. 2a, c), while when deposited at 260 °C, many agglomerates, sized around 1 µm are present (Fig. 2b, d). Hafnia tends to form bigger agglomerates at 260 °C compared to alumina, mainly due to the formation of a crystalline phase with increasing temperature, which we observed in our previous work [40], and was previously demonstrated by other groups [62]. Further, the rough morphology at the nanoscale is a characteristic feature of the ALD hafnia thin films (insets in Fig. 2). These tiny agglomerates, less than 100 nm in size, are an indication of 3D growth, so the film has a columnar structure. However, this characteristic of hafnia films is dependent on the deposition temperature. At 260 °C, a higher density of these agglomerates can be observed (insets in Fig. 2b, d) compared to deposition at 180 °C (insets in Fig. 2a, c).

### Wettability and surface energy

**Fig. 3:**
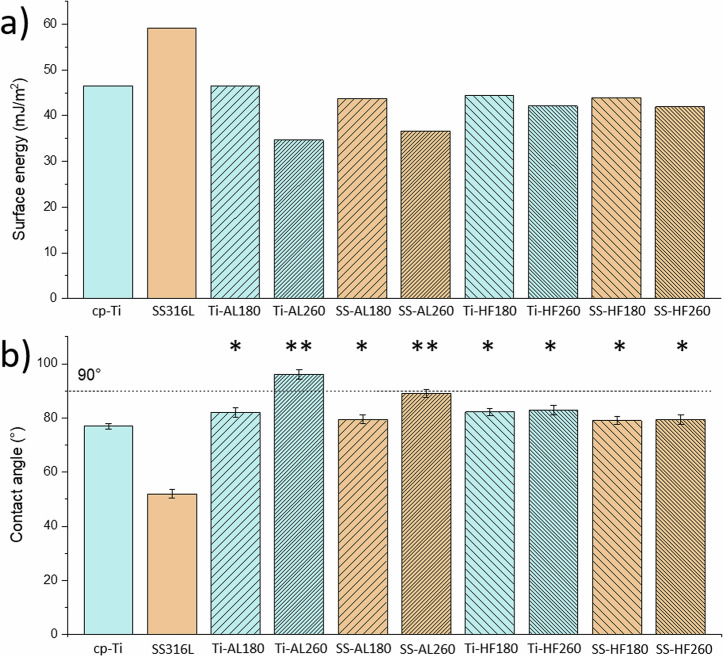
Results of wettability measurements, **a** total surface energies for all specimens, **b** contact angles of water drops. Statistically significant differences relative to bare metals, cp-Ti and SS316L, are indicated as *p < 0.001; and Ti-AL260 and SS-AL260 relative to the other samples are indicated as **p < 0.0001

The obtained surface energy results show the difference between the surfaces of cp-Ti and SS316L, as the latter has better wettability (smaller contact angle) and higher surface energy (Fig. 3). The result for pure cp-Ti with a contact angle of 77° agrees with the literature data [10]. Polished SS316L specimens are more hydrophilic than polished Ti specimens, with a contact angle of 52°.

ALD-coated 316L specimens show a higher contact angle (lower surface energy) than the bare sample, whereas the difference to the bare sample is not so pronounced for cp-Ti. cp-Ti and SS316L specimens coated with alumina at 180 °C and hafnia at 180 °C or 260 °C showed almost the same wettability as bare cp-Ti, while specimens with alumina deposited at 260 °C showed a deviation. Namely, Ti-AL260 and SS-AL260 specimens exhibit significantly lower surface energy compared to all other specimens (Fig. 3a). Also, considering the increased values of the water contact angles for these two specimens (Fig. 3b), it can be concluded that alumina deposited at a higher temperature tends to be hydrophobic. Interestingly, this is not a consequence of the surface morphology, i.e. due to the small densely distributed agglomerates below 1 µm in size (Fig. 1c, d); otherwise, SS-AL180 would also show an increased contact angle and decreased surface energy. Therefore, the reason must lie in the chemical properties of the alumina surface, although surface morphology and surface chemistry equally determine wettability [68]. This issue is further discussed in Section 3.4. related to antibacterial properties.

### Biocompatibility properties

Mesenchymal stem cells are multipotent, potentially originating osteo-, chondro- and adipogenic- lineages, making them extremely important in tissue regeneration/repair processes [69], particularly in musculoskeletal applications. Here, the biocompatibility of the prepared materials was evaluated in vitro using human mesenchymal stem cells (hMSCs) with two different assays.

**Fig. 4:**
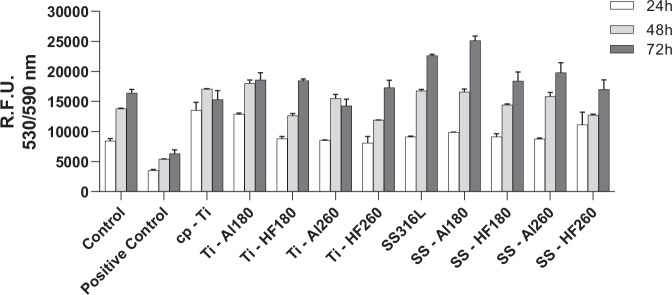
Results of resazurin assay showing hMSCs metabolic activity after culturing in specimen extracts and control media for 24 h, 48 h and 72 h. The positive control (cytotoxic) consisted of cells treated with triton X-100. Data represents the mean ± SD of 3 technical replicates of one representative experiment

**Fig. 5:**
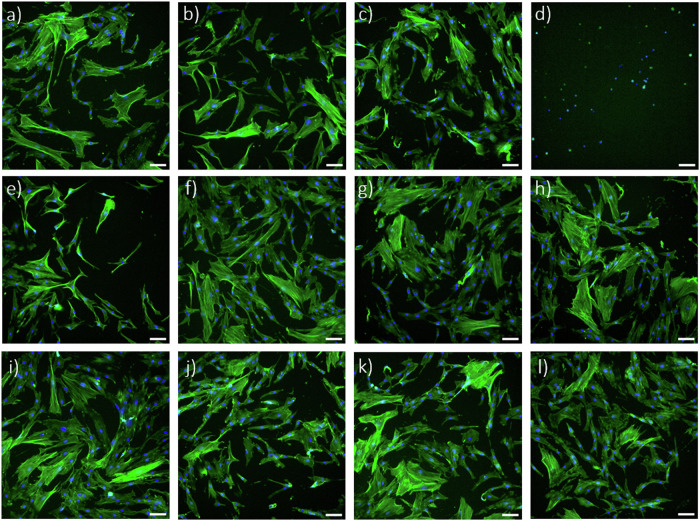
Fluorescent labelling of F-actin (green) and nuclei (blue) of hMSCs cultured for 72 h in extracts of tested specimens and in control media; **a** control containing fresh culture medium, **b** cp-Ti extract **c** SS316L extract, **d** positive control or culture medium with added Triton X-100, **e** Ti-AL180 extract, **f** Ti-HF180 extract, **g** Ti-AL260 extract, **h** Ti-HF260 extract, **i** SS-AL180 extract, **j** SS-HF180 extract, **k** SS-AL260 extract and **l** SS-HF260 extract. Scale bar = 100 μm

The first method consisted of treating cells with extracts of each specimen and quantifying their metabolic activity as a function of time to assess the effect of releasing any cytotoxic species from the ALD thin films. The results of the resazurin assay show that cell metabolic activity increases with time in culture, suggesting no cytotoxic effect of any of the extracts (Fig. 4). Additionally, the results of all extracts are comparable with the untreated cells (negative control) and significantly different (*p* < 0.0001, for all specimens and all time points) from cells treated with triton X-100, a powerful detergent, added to the cell culture medium to cause cell membrane damage and, consequently, cell death (positive control). At the end of the culture period (72 h), filamentous actin and nuclei were stained to assess cell morphology. Representative fluorescence microscopy images show that cellular morphology is similar in all conditions (Fig. 5a–c and e–l), except for the positive control (Fig. 5d), where only small pyknotic nuclei are observable. Thus, this indicates that none of the tested specimens releases harmful species during incubation that would interfere with cell development, i.e. they all have potentially good biocompatibility.

**Fig. 6:**
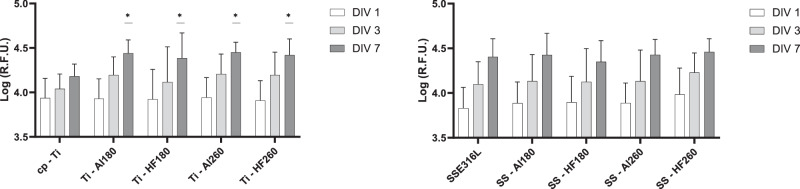
Results of Resazurin assays showing hMSCs viability after culturing directly on the specimen surface after 1, 3 and 7 days. Data represents the mean ± SD of 3 independent experiments. *p < 0.05 in relation to cp-Ti

**Fig. 7:**
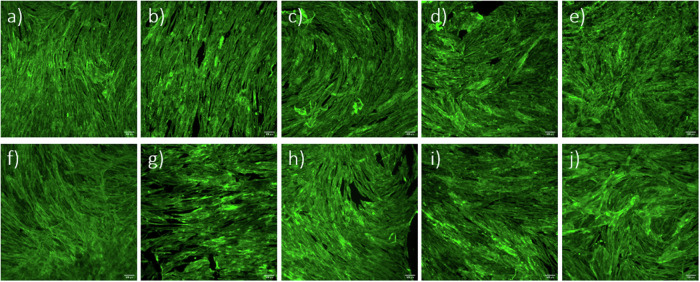
Fluorescent labelling of F-actin (green) of hMSCs cells cultured for 7 days directly on tested specimens; **a** cp-Ti, **b** Ti-AL180, **c** Ti-HF180, **d** Ti-AL260, **e** Ti-HF260, **f** SS316L, **g** SS-AL180, **h** SS-HF180, **i** SS-AL260, and **j** SS-HF260. Scale bar = 100 μm

Based on the positive results obtained, we proceeded with the direct seeding of hMSCs on the surfaces of the tested specimens. In this case, cells were maintained in culture for 7 days, with cell metabolic activity being measured at days 1, 3 and 7 in vitro (Fig. 6). Results show a significant increase in metabolic activity at day 7 for all coated cp-Ti specimens when compared to bare cp-Ti. SS316L specimens did not show a significantly altered metabolic activity after applying ALD thin films. Fluorescence microscopy images show no observable differences in cellular morphology in all specimens (Fig. 7). This data indicates that ALD modifications are not detrimental and even result in a better outcome than for bare cp-Ti. These results also suggest that surface morphology, i.e. the presence and size of agglomerates, nor different wettability, do not affect the viability of hMSCs, as might be expected based on previous works [59–61]. Namely, there were no significant differences in cell viability between hafnia thin films deposited at different temperatures even though they show distinctively different surface morphologies (Fig. 2). Further, biocompatibility results for alumina thin films deposited at 260 °C, which showed lower surface energies (higher contact angle) (Fig. 3), did not differ from other specimens.

### Antibacterial properties

**Fig. 8:**
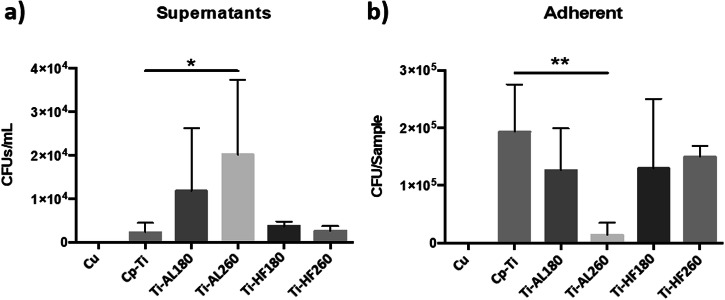
Antibacterial activity of cp-Ti and cp-Ti-coated surfaces against *S. aureus* after incubation for 2 h. Results for incubation media and adherent bacteria are the average of two independent experiments with three replicates each. Data is expressed as the mean ± standard deviation (SD). Statistically significant differences relative to bare cp-Ti are indicated as *p < 0.05; **p < 0.01

Antibacterial properties were determined by direct contact of *Staphylococcus aureus* with the specimen surfaces. Colony-forming units were counted in the incubation medium and on sonicated samples (surface-adhered bacteria). Pure Cu, a known bactericide, was used as a positive control. SEM imaging was also performed for visual insight into adhered bacteria. Results for bare and coated cp-Ti specimens and Cu positive control regarding CFU in the incubation medium and adhered bacteria on the specimen surfaces are shown in Fig. 8. No live bacteria were present on the Cu specimen either in the incubation medium or adhered to the surface. On bare cp-Ti, significantly more bacteria remained on the specimen surface, i.e. adhered (Fig. 8b), than in the incubation medium (Fig. 8a). The same behaviour is valid for Ti-AL180. Ti-AL260 shows an anti-adhesive effect, as demonstrated by the statistically significant reduction of adherent bacteria and increased CFU in the incubation media. This can be attributed to the low wettability, i.e. reduced surface energy of Ti-AL260 (Fig. 3b). In general, bacteria with hydrophobic cell surfaces favour hydrophobic material surfaces, while those with hydrophilic cell surfaces favour hydrophilic material surfaces [70]. Therefore, hydrophobic alumina deposited at a higher temperature appears to be a non-attractive substrate for hydrophilic bacteria. As the Ti-AL180 specimen does not show a similar effect despite that both specimens have similar morphology (Fig. 1a, b), it can be concluded that wettability and chemical properties could be critical for inhibiting of *S. aureus* adhesion. Hafnia-coated specimens Ti-HF180 and Ti-HF260 also showed significantly more adhered bacteria on the surface than in the incubation medium, as observed for cp-Ti and Ti-AL180.

Since the surfaces were sterilised using ethanol (as described in Section 2.5) before exposure to bacteria, the contact angles were measured before and after sterilisation to exclude its effect on the surface properties. The results for sterilised alumina-coated samples showed no change in the contact angles compared to those shown in Fig. 3b for deposition temperatures of 180 and 260 °C. In other words, the surface chemistry remained stable, with a more hydrophobic surface deposited at 260 °C. The latter seems less favourable for bacteria to adhere (Fig. 8b). In contrast, specimens coated with hafnia thin films showed fairly uniform results regardless of the substrate and the deposition temperature (Fig. 3). However, these samples became almost perfectly hydrophilic after sterilisation, so that the contact angle could not even be measured. Therefore, their surface chemistry has completely changed, and the surface energy has increased enormously, thus preferably the surface for the strain of *S. aureus* bacteria. Besides, although Ti-HF180 and Ti-HF260 have distinctively different surface morphology (Fig. 2a, b), with the latter showing a more agglomerated surface, the attachment of the bacteria was similar.

**Fig. 9:**
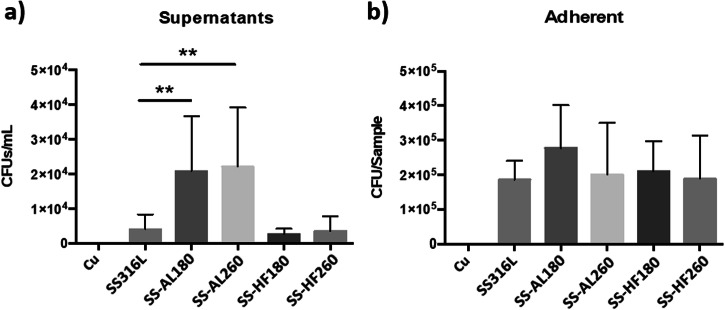
Antibacterial activity of SS316L and SS316L-coated surfaces against *S. aureus* after incubation for 2 h. Results for incubation media and adherent bacteria are the average of three independent experiments with three replicates each. Data is expressed as the mean ± standard deviation (SD). Statistically significant differences relative to bare SS316L are indicated as **p < 0.01

The antibacterial activity of the SS316L specimens proved to be slightly different to that of cp-Ti (Fig. 9). Bare SS316L showed similar interactions with bacteria as cp-Ti, i.e. more bacteria remained adhered to the surface than in the incubation medium. SS-AL180 and SSAL260 samples presented a statistically significant increase of CFU in the incubation medium, although the expected reduction in the number of adherent bacteria was not observed. Therefore, SS-AL260 did not show the expected result comparable to Ti-Al260, i.e. anti-adhesive property due to low wettability. Indeed, SS-AL260 wettability and surface energy show results slightly different from Ti-AL260, i.e. a slightly lower wettability and a slightly higher surface energy (Fig. 3), respectively. Perhaps its surface energy did not reach the critically low point that would enable the anti-adhesion as in the case of Ti-AL260 (Fig. 8b). SS-HF180 and SS-HF260 show the same properties as Ti-HF180 and Ti-HF260, and SS316L, i.e. preferred adhesion of bacteria to the surfaces with less remaining in the incubation medium. Therefore, the possible hypothesis that small densely distributed agglomerates on the surface of the alumina film can act as alumina nanoparticles, i.e. have a bactericidal role [51], seems unlikely. However, it appears that the surface chemistry controls the surface energy and, thus, the adhesion of the *S. aureus* bacteria.

**Fig. 10:**
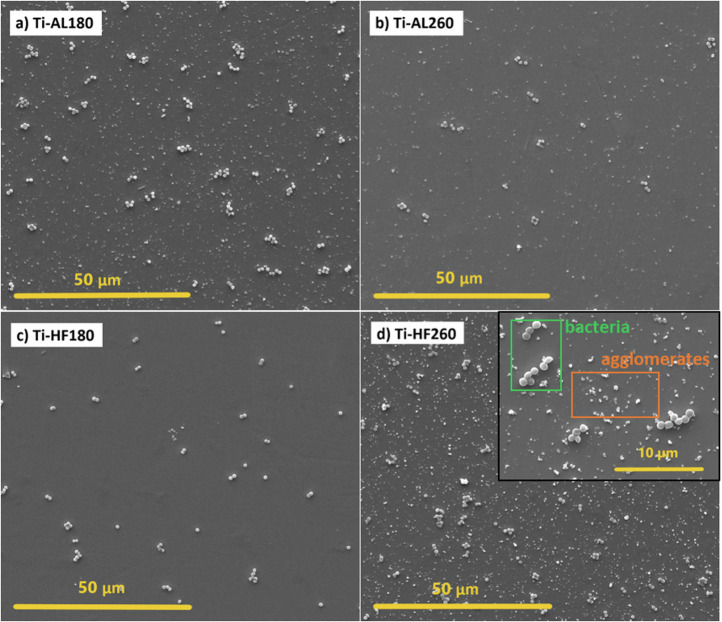
SEM images of ALD alumina- and hafnia-coated cp-Ti specimens after incubation for 2 h with the *S. aureus* for **a** alumina deposited at 180 °C, **b** alumina deposited at 260 °C, **c** hafnia deposited at 180 °C and **d** hafnia deposited on 260 °C. White round bright clusters are adhered bacteria, while smaller white deposits are agglomerates

SEM images of the visual illustration of the adhesion of bacterial colonies on the ALD-coated cp-Ti specimens are shown in Fig. 10. SEM results are only complementary results for visual information and should not be taken as quantitative. *S. aureus* is a round-shaped bacteria that usually form clumps as bright white clusters, as detailed in the inset in Fig. 10d. Regardless of the similar morphology of Ti-AL180 and Ti-AL260 (Fig. 1a, b), visually, fewer bacteria can be observed on the surface of Ti-AL260 (Fig. 10a, b) that could be related to its low surface energy (Fig. 3b). This is in good correlation with the results of CFU counting **(**Fig. 8).

On the other hand, Ti-HF180 and Ti-HF260, despite a significant difference in morphology (Fig. 2a, b), i.e. the absence of agglomerates in Ti-HF180, the presence of bacteria observed in both cases was similar (Fig. 10c, d). Note that clusters of adhered bacteria should be distinguished from agglomerates formed at 260 °C (inset in Fig. 10d).

## Conclusions

Two types of thin films, alumina and hafnia, were deposited by atomic layer deposition on commercially pure titanium and stainless steel 316 L at 180 °C and 260 °C aiming to investigate the effect of film composition, surface morphology and surface energy on their biocompatibility and antibacterial properties. For hafnia, the effect of temperature is related to forming more agglomerates, whereas, for alumina, this effect was not so pronounced. Surface energy was the lowest for the Ti coated with alumina at 260 °C. The favourable surface morphology of implant materials, i.e. roughness on micro and sub-micro scale, is crucial for good interaction between implanted material and tissue.

Different surface morphologies on a micro-scale, primarily the presence of agglomerates, did not affect the biocompatibility of the ALD-coated specimens. Also, the different surface energies, i.e. their wettability, were not decisive parameters of biocompatibility tests regardless of the deposition temperature. ALD alumina and hafnia did not produce a harmful effect on human bone marrow-derived mesenchymal stem cells. Moreover, a statistically significant increase in metabolic activity after 7 days was observed for all ALD-coated cp-Ti specimens compared to bare cp-Ti when hMSCs were seeded directly on the specimens’ surface. No increase was observed for SS316L specimens.

Regarding the antibacterial properties, no bactericidal effect on *S. aureus* was found on any specimen. Generally, bacterial adhesion shows no correlation with surface morphology, but surface energy has a particular effect on the adhesion of bacteria. ALD alumina thin film deposited on cp-Ti at 260 °C showed anti-adhesive properties to bacteria, which we ascribed to the decreased surface energy measured for this specimen. SEM confirmed a low number of adherent bacteria. It seems that the different nature of the substrate affects the growth and properties of the alumina ALD film differently, as similar effect was not observed for the film on 316L specimen.

Results prove that *ca*. 60 nm thick alumina and hafnia films produced by atomic layer deposition do not impair the biocompatibility of biomedical alloys. Moreover, an even better outcome was observed for ALD-coated cp-Ti compared to the bare substrate. Similarly, anti-adhesive properties shown by ALD alumina films on cp-Ti deposited under optimal conditions offer additional functionality when considering implantation in the human body. The general conclusion is that ALD-coated biomedical alloys are potentially applicable in biomedical applications and worth further investigation.

## Data Availability

The data that support the findings of this study are available on request from the corresponding author.
